# Effects of topiroxostat on the serum urate levels and urinary albumin excretion in hyperuricemic stage 3 chronic kidney disease patients with or without gout

**DOI:** 10.1007/s10157-014-0935-8

**Published:** 2014-01-22

**Authors:** Tatsuo Hosoya, Iwao Ohno, Shinsuke Nomura, Ichiro Hisatome, Shunya Uchida, Shin Fujimori, Tetsuya Yamamoto, Shigeko Hara

**Affiliations:** 1 Department of Pathophysiology and Therapy in Chronic Kidney Disease, Jikei University School of Medicine, 3-25-8, Nishi-Shimbashi, Minato-ku, Tokyo, 105-8461 Japan; 2 Division of General Medicine, Department of Internal Medicine, Jikei University School of Medicine, Minato-ku, Tokyo, Japan; 3 Kidney Center Nephrology and Dialysis Unit, Suzuka Kaisei Hospital, Suzuka, Mie Japan; 4 Division of Regenerative Medicine and Therapeutics, Institute of Regenerative Medicine and Biofunction, Tottori University Graduate School of Medical Science, Yonago, Tottori Japan; 5 Department of Internal Medicine, Teikyo University School of Medicine, Itabashi-ku, Tokyo, Japan; 6 Division of Endocrinology and Metabolism, Department of Internal Medicine, Hyogo College of Medicine, Hyogo, Japan; 7 Center of Health Management and Okinaka Memorial Institute for Medical Research, Toranomon Hospital, Minato-ku, Tokyo, Japan

**Keywords:** Hyperuricemia, Gout, CKD, Topiroxostat, FYX-051

## Abstract

**Background:**

Topiroxostat, a selective xanthine oxidase inhibitor, shows effective reduction in the serum urate level in hyperuricemic patients with or without gout. The objective of this study was to evaluate the efficacy and safety of topiroxostat in hyperuricemic stage 3 chronic kidney disease patients with or without gout.

**Methods:**

The study design was a 22-week, randomized, multicenter, double-blind study. The enrolled patients were randomly assigned to treatment with topiroxostat 160 mg/day (*n* = 62) or to the placebo (*n* = 61). The endpoints were the percent change in the serum urate level, change in the estimated glomerular filtration rate, the urinary albumin-to-creatinine ratio, the proportion of patients with serum urate levels of 356.88 μmol/L or less, blood pressure, and serum adiponectin.

**Results:**

After 22 weeks, although the changes in the estimated glomerular filtration rate and blood pressure were not significant, the percent change in the serum urate level (−45.38 vs. −0.08 %, *P* < 0.0001) and the percent change in urinary albumin-to-creatinine ratio (−33.0 vs. −6.0 %, *P* = 0.0092) were found to have decreased in the topiroxostat as compared with the placebo. Although the incidence of ‘alanine aminotransferase increased’ was higher in the topiroxostat, serious adverse event rates were similar in the two groups.

**Conclusion:**

Topiroxostat 160 mg effectively reduced the serum urate level in the hyperuricemic stage 3 chronic kidney disease patients with or without gout.

## Introduction

Chronic kidney disease (CKD) is one of the major comorbidities in patients with gout and hyperuricemia [1]. The relationship between the onset or progression of CKD and hyperuricemia has been widely examined in observational trials, and hyperuricemia has come to be recognized as a risk factor for renal failure in the general population in Japan [2–5]. In addition, elevated serum urate has been reported to be associated with an increase in the risk for hypertension, cardiovascular diseases, and metabolic diseases [6–8]. However, whether hyperuricemia plays a role in the pathogenesis of these disease states or is just a marker of the disease states still remains controversial [9]. Thus, intervention studies for ameliorating hyperuricemia or gout are expected to play more important roles in elucidating these important clinical issues. Intervention studies of allopurinol, which decreases serum urate levels by inhibiting xanthine oxidase, have shown a renoprotective effect in patients with gout and CKD [10, 11]. These findings are clinically important, especially in the context of increasing prevalence of end-stage renal disease in the general population [12]. However, there are a few reports that have confirmed the renoprotective effect of allopurinol in patients with CKD, and it remains unclear whether the renoprotective effect of the drug might originate from the reduction of the serum urate level, allopurinol itself, or the inhibition of xanthine oxidase. Thus, we considered it clinically important to conduct intervention studies with other urate-lowering agents.

Topiroxostat (formerly known as FYX-051) is an orally administered non-purine analog, selective xanthine oxidase (XO) inhibitor developed for the management of hyperuricemia, including in patients with gout, in Japan. Unlike structure-based XO inhibitors, such as febuxostat, topiroxostat not only interacts with amino acid residues of the solvent channel, but also binds covalently to molybdenum via oxygen in the hydroxylation reaction intermediate [13, 14]. In addition, the pharmacokinetics of neither unchanged topiroxostat nor of its metabolites is affected by mild-to-moderate renal impairment (unpublished data).

In the treatment of hyperuricemia and gout, XO inhibitors such as allopurinol or febuxostat are considered to be first-line drugs [15]. However, in a view of safety concern, the reduction of allopurinol dose is recommended in patients with renal impairment; furthermore, the urate-lowering efficacy of allopurinol is inadequate to control hyperuricemia in patients with gout [16–19]. On the other side, febuxostat has been shown to exhibit urate-lowering efficacy in patients with renal impairment [20]. However, the usage experience of febuxostat in CKD patients is still insufficient [21].

The objective of this multicenter, double-blind, randomized placebo-controlled study was to evaluate the effect of topiroxostat in reducing the serum urate level, and to improve the estimated glomerular filtration rate (eGFR), urinary albumin-to-creatinine ratio (ACR), blood pressure, and serum adiponectin levels in hyperuricemic patients with renal impairment, with or without gout.

## Methods

The protocol and informed consent form were reviewed and approved by the institutional review board at each study center. This study was conducted in compliance with the Declaration of Helsinki (1996 version), Good Clinical Practice guidelines and other applicable regulatory requirements. Written informed consent was obtained from all trial subjects before conducting of any study-specific procedures. The information of this study was registered to the Japan Pharmaceutical Information Center (JAPIC) on June 28, 2010 (Registration Number: JapicCTI-101171).

### Study design, study population and treatment

This study was a 22-week, multicenter, randomized, double-blind, placebo-controlled study carried out in Japan to assess the efficacy and tolerability of topiroxostat in hyperuricemic patients with renal impairment, with or without gout. Eligible patients were men or women aged 20–75 years, with hyperuricemia (defined as serum urate levels >475.84 μmol/L, or serum urate levels >416.36 μmol/L in patients with gout), and eGFR of ≥30 to <60 mL/min/1.72 m^2^ within the preceding 3 months. The exclusion criteria were: onset of gouty arthritis within 2 weeks prior to the start of the study (baseline); nephrotic syndrome; renal function impairment associated with nephrolithiasis or urolithiasis; change of the serum creatinine level by more than 44.2 μmol/L per month within the 8-week run-in period; hyperuricemia possibly secondary to a malignant tumor or other diseases; HbA1c ≥8.0 %; severe hypertension (SBP ≥180 mmHg or DBP ≥110 mmHg); hepatic dysfunction (AST or ALT ≥100 IU/L); cancer; pregnancy; breastfeeding; serious hepatic disease; serious heart disease; any other significant medical conditions.

In the eligible patients who were enrolled in the study, the current urate-lowering therapy was withdrawn with informed consent before at least 2 weeks prior to the initiation of an 8-week run-in period. At the end of the run-in period, the patients were assigned (1:1) to either the topiroxostat 160 mg/day group or the matching placebo group at the central registration center.

Topiroxostat (or matching placebo) was administered orally at an initial dose of 40 mg/day for 2 weeks, followed in a stepwise manner by increase of the dose to 80 mg/day for 4 weeks, 120 mg/day for 8 weeks, and 160 mg/day for 8 weeks. All agents which could potentially affect the serum urate level were discontinued during the study. Because of assessment of the incidence of gouty arthritis in the study, we did not permit colchicine prophylaxis during the study. When gouty arthritis occurred during the study, colchicine, NSAIDs, or corticosteroids were used to treat the gouty arthritis at the investigator’s discretion. Using antihypertensive agents and antihyperlipidemic agents were restricted during the study. The dose and type of these drugs were maintained as far as possible after randomization.

To maintain the double-blind condition, serum urate levels measured after administration of the study drug in each patient were concealed from both the investigators and the patients throughout the study period.

### Endpoints

The primary endpoints were the percent change of the serum urate level from the baseline to the final visit and change of the eGFR from the baseline to the final visit. The secondary efficacy endpoints were the percent change of the ACR from the baseline to the final visit, changes in the home blood pressure from baseline to the final visit, proportion of patients with serum urate levels ≤356.88 μmol/L at the final visit, and change of the serum adiponectin level from baseline to the final visit. The ACR was calculated from the levels of albumin and creatinine in the urine sample taken at hospital.

The safety and tolerability of study drug treatment were assessed by physical examinations, vital signs measurements, laboratory tests, and adverse event (AE) monitoring. All laboratory tests were performed at a centralized lab. UA-767PC (A&D Company, Limited, Tokyo, Japan) was used for measurements of the home blood pressure values.

### Statistical methods

We referred to the result of intervention study of allopurinol for information about the sample size of this study [10]. We calculated that 53 patients per group were needed to detect an absolute difference in the serum creatinine level of 26.52 ± 41.5 μmol/L from the baseline to the final measurement between the topiroxostat group and placebo group at a two-sided significance level of 0.05, with at least 90 % power. Taking into consideration possible dropout of some patients, we set the required number of patients in each group at 60. Efficacy analyses were performed using the intent-to-treat population, which included all randomized patients who had received at least one dose of the study medication during the study and in whom at least one post-baseline efficacy measurement had been made. For patients who dropped out of the study, the missing data were complemented by the last observation carry-forward method. The data were expressed as mean ± SD for continuous normally distributed variables, and as geometric means and interquartile ranges for non-normally distributed variables.

The baseline characteristics are summarized by treatment group using appropriate descriptive statistics. The *χ* ^2^ test or Fisher’s exact test for categorical variables and Student’s *t* test for continuous variables were used to test for homogeneity between the treatment groups at baseline.

As for the efficacy analyses, comparisons of the mean values were performed using the Student’s *t* test or paired *t* test. To avoid multiplicity of the primary endpoints, a 2-step closed testing procedure was planned. First, comparison of the percent change of the serum urate level from the baseline to the final visit between the groups was carried out. Second, if the result of the first step test was statistically significant, comparison of the change of the eGFR from the baseline to the final visit between the groups was carried out. As the ACR and serum adiponectin showed a skewed distribution, raw values were log-transformed for calculation and the geometric mean ratios from the baseline were calculated. For simultaneous assessment of the effect of treatment on the changes in the eGFR from the baseline after adjustments for covariates (eGFR, ACR and HbA1c at baseline), an analysis of covariance models on the eGFR was used. Similarly, for that after adjustment for the covariate of baseline ACR, an analysis of covariance models on the log-transformed ACR was used. A correlation analysis was performed using Pearson’s correlation test.

Safety analyses were performed using the safety population, which included all randomized patients who had received at least one dose of the study drug. The incidences of adverse events (AEs) are summarized by the primary organ system involved, the preferred name, severity, and causal relationship to the study drug. The incidence of death, other serious AEs, and the AEs leading to study discontinuation are also summarized.

Analyses were performed using the SAS statistical software, version 9.1 (SAS Institute, Cary, NC), with the Windows operating system. Statistical tests for baseline characteristics were two-sided and *P* values ≤0.15 were considered to denote statistical significance. The other statistical tests and confidence intervals were 2-sided and *P* values ≤0.05 were considered to be statistically significant.

## Results

### Patient population

**Fig. 1:**
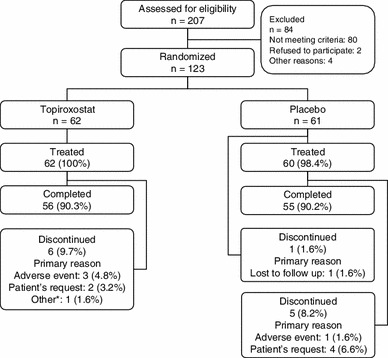
Patient distribution. *Asterisk* discontinuance criteria (serum urate <118.96 μmol/L)

Of the 207 patients who were screened, 123 (topiroxostat group 62, and placebo group 61) were randomized to the treatment groups. Among the randomized patients, one patient from placebo group was not treated with the study drug. Therefore, the safety population included 122 patients (topiroxostat group 62, and placebo group 60). Eleven patients (topiroxostat group 6 patients, placebo group 5 patients) were withdrawn from the study, primarily due to the appearance of AEs or at the patient’s request. 62 patients from the topiroxostat group and 60 patients from the placebo group were included in the intent-to-treat population (Fig. 1). Among intent-to-treat population, the serum urate was not measured in two patients of the topiroxostat group at the point of discontinuation of the study.

The baseline characteristics of the two treatment groups were similar, except for a lower proportion of patients with complication of diabetes in the topiroxostat group (29.0 vs. 41.7 %; *P* = 0.1442) (Table 1).

**Table 1:** Summary of the baseline characteristics of the intent-to-treat population

Variable	Topiroxostat (*n* = 62)	Placebo (*n* = 60)	*P* value
Age (years)	62.5 ± 8.8	64.6 ± 8.1	0.1850^3^
Sex (male/female)	53/9	56/4	0.1600^1^
Body mass index (kg/m^2^)	25.75 ± 4.45	25.51 ± 3.10	0.7203^3^
Serum urate (μmol/L)	503.80 ± 73.76	503.80 ± 76.13	0.9968^3^
Duration of hyperuricemia (years)	9.65 ± 11.23	9.51 ± 9.24	0.9472^3^
Diabetic nephropathy, n (%)	14 (22.6)	19 (31.7)	0.2587^1^
Chronic glomerulonephritis, *n* (%)	3 (4.8)	5 (8.3)	0.4875^2^
Nephrosclerosis, *n* (%)	10 (16.1)	12 (20.0)	0.5782^1^
Diabetes, *n* (%)	18 (29.0)	25 (41.7)	0.1442^1^
eGFR (mL/min/1.73 m^2^)	49.40 ± 8.93	48.89 ± 8.51	0.7434^3^
ACR (mg/g) geometric mean (IQR)	41.71 (12.53–132.70)	29.92 (11.05–48.15)	0.2341^3^
SBP (mmHg)	135.2 ± 17.3	134.6 ± 20.0	0.8603^3^
DBP (mmHg)	84.8 ± 11.8	84.1 ± 11.6	0.7476^3^
Serum Adiponectin (μg/mL)	9.29 ± 5.47	10.30 ± 6.45	0.3559^3^
RAA blockers, *n* (%)	38 (61.3)	31 (51.7)	0.2837^1^

*eGFR* estimated glomerular filtration rate, *ACR* urinary albumin-to-creatinine ratio, *SBP* systolic blood pressure, *DBP* diastolic blood pressure, *RAA* *blockers* use of angiotensin II receptor blockers, angiotensin-converting enzyme inhibitors, aldosterone blockers, or renin inhibitor

^1^ *χ* ^2^ test, ^2^ Fisher’s exact test, ^3 ^Student’s *t* test

### Percent change of the serum urate

**Fig. 2:**
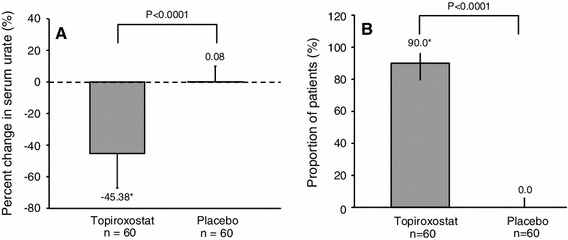
Percent change of the serum urate levels and proportion of patients with serum urate levels ≤356.88 μmol/L at the final visit (intent-to-treat population). **a** Percent change of the serum urate level from the baseline to the final visit. Results are expressed as mean ± SD. **b** Proportion of patients with serum urate levels ≤356.88 μmol/L at the final visit. Results are expressed as percentages and its 95 % CIs. Two patients of the topiroxostat group were withdrawn without measurement of the serum urate levels during the study. Therefore, these patients were excluded from this analysis. *SD* standard deviation, *CI* confidential interval

The percent change of the serum urate from the baseline to the final visit was significantly higher in the topiroxostat group than that in the placebo group (topiroxostat: −45.38 ± 21.80 % (*n* = 60), placebo: 0.08 ± 9.92 % (*n* = 60), between-group difference: −45.46 %; 95 % confidence interval (CI) −39.33 to −51.58, *P* < 0.0001) (Fig. 2a).

### Estimated glomerular filtration rate

**Fig. 3:**
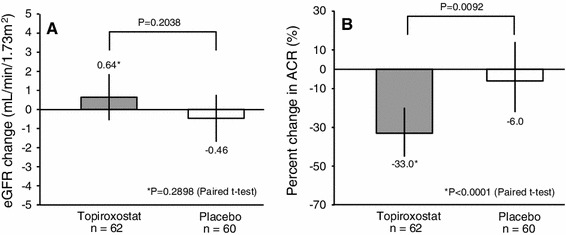
Change of the eGFR and ACR from the baseline to the final visit (intent-to-treat population). **a** Changes of the eGFR from the baseline to the final visit. Results are expressed as point estimates and its 95 % CIs by ANCOVA. Covariates: baseline eGFR, baseline ACR, baseline HbA1c. **b** Percentage of the ACR from the baseline to the final visit. Results are expressed as point estimates and its 95 % CIs as calculated by ANCOVA. Covariate: baseline ACR. *eGFR* estimated glomerular filtration rate, *ACR* urinary albumin-to-creatinine ratio, *SD* standard deviation, *CI* confidential interval, *ANCOVA* analysis of covariance

**Fig. 4:**
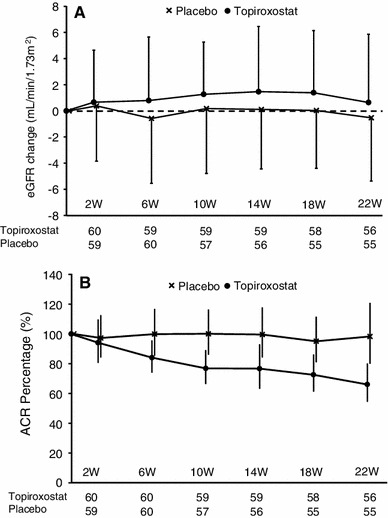
Changes of the eGFR and ACR from the baseline to each visit (intent-to-treat population). **a** Changes of the eGFR from the baseline to each visit. Results are expressed as mean ± SD. **b** Percent changes of the ACR from the baseline to each visit. Results are expressed as means and its 95 % CIs. *eGFR* estimated glomerular filtration rate, *ACR* urinary albumin-to-creatinine ratio, *SD* standard deviation, *CI* confidential interval

The change of the eGFR from the baseline to the final visit tended to be higher in the topiroxostat group as compared to that in the placebo group as analyzed by analysis of covariance (ANCOVA), however, the difference was not statistically significant (topiroxostat: 0.64 mL/min/1.73 m^2^; 95 % CI −0.55 to 1.84, placebo: −0.46 mL/min/1.73 m^2^; 95 % CI −1.68 to 0.75, between-group difference: 1.10 mL/min/1.73 m^2^; 95 % CI −0.61 to 2.82, *P* = 0.2038) (Fig. 3a). The changes in the eGFR from the baseline to each visit are shown in Fig. 4a.

### Achievement rate of serum urate levels

The proportion of patients with serum urate levels ≤356.88 μmol/L at the final visit was higher in the topiroxostat group than that in the placebo group (topiroxostat: 90.0 %; 95 % CI 79.5–96.2 % (*n* = 60), placebo: 0.0 %; 95 % CI 0.0–6.0 %; *P* < 0.0001) (Fig. 2b).

### Urinary albumin-to-creatinine ratio

**Fig. 5:**
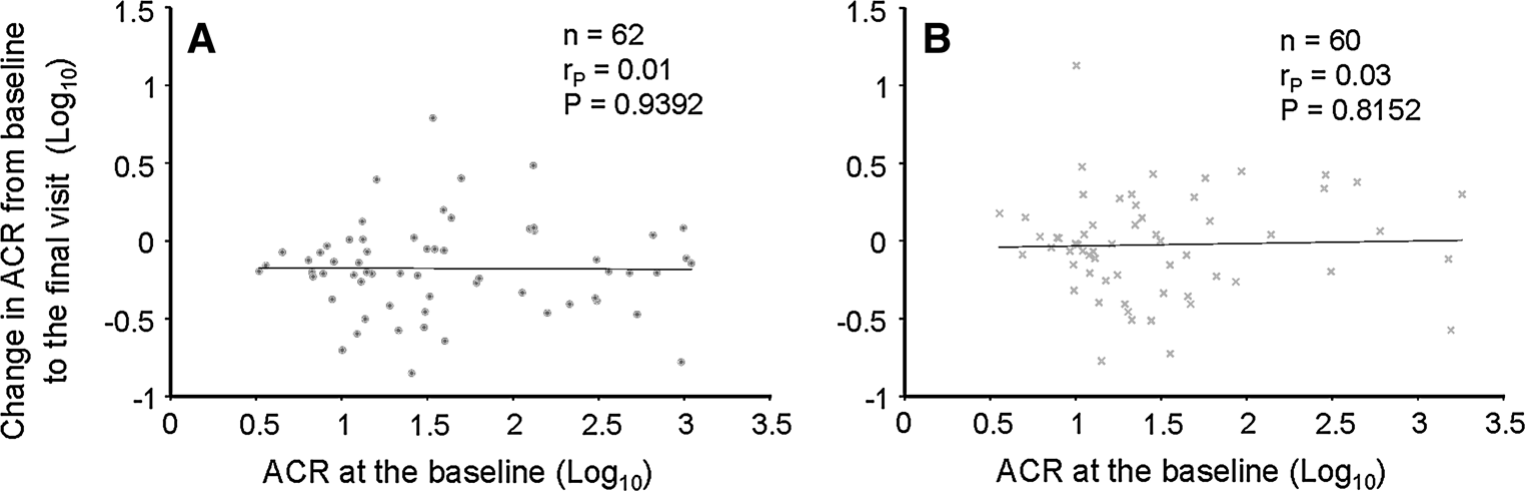
Correlation between the baseline ACR and the change in the ACR from the baseline to the final visit in each group. **a** Topiroxostat group (*n* = 62). **b** Placebo (*n* = 60)*. ACR* Urinary albumin-to-creatinine ratio, *r*_*P*_ Pearson’s correlation coefficient

The percent change of the ACR from the baseline to the final visit was higher in the topiroxostat group than that in the placebo group as analyzed by ANCOVA (topiroxostat: −33.0 %; 95 % CI −45.0 to −20.0 %, placebo: −6 %; 95 % CI −22.0 to 14.0 %; *P* = 0.0092) (Fig. 3b). The trend of the percent change of the ACR from the baseline is shown in Fig. 4b. The change in the ACR from the baseline to the final visit was not correlated with the baseline ACR in either group (Fig. 5).

### Home blood pressure

There were no significant differences in the degree of reduction of the systolic blood pressure (SBP) or diastolic blood pressure (DBP) between the topiroxostat group and placebo group (change in SBP from baseline to the final visit: −1.7 ± 18.3 mmHg (*n* = 62) vs. −0.3 ± 21.1 mmHg (*n* = 59); *P* = 0.6963; change in DBP from baseline to the final visit: −2.5 ± 10.3 mmHg (*n* = 62) vs. −2.7 ± 12.3 mmHg (*n* = 59); *P* = 0.9058) (Table 2).

**Table 2:** Systolic and diastolic blood pressure levels at the baseline and during follow-up (intent-to-treat population)

	SBP (mmHg)		DBP (mmHg)	
	Topiroxostat	Placebo	Topiroxostat	Placebo
Baseline	135.2 ± 17.3 (62)	134.6 ± 20.0 (59)	84.8 ± 11.8 (62)	84.1 ± 11.6 (59)
Week 2	134.2 ± 18.3 (60)	136.3 ± 21.0 (59)	84.8 ± 11.9 (60)	83.7 ± 11.7 (59)
Week 6	133.3 ± 18.0 (60)	132.5 ± 20.8 (60)	84.3 ± 10.7 (60)	82.8 ± 12.4 (60)
Week 10	132.1 ± 16.4 (60)	134.1 ± 22.3 (57)	82.8 ± 11.8 (60)	82.2 ± 12.9 (57)
Week 14	131.9 ± 19.5 (59)	131.3 ± 20.0 (55)	82.6 ± 11.5 (59)	80.5 ± 10.4 (55)
Week 18	131.5 ± 18.4 (58)	131.6 ± 20.3 (54)	81.6 ± 11.1 (58)	80.2 ± 10.9 (54)
Week 22	133.6 ± 17.8 (56)	133.8 ± 21.2 (55)	81.7 ± 11.6 (56)	80.9 ± 10.4 (55)

Mean ± SD (*n*)

*SBP* systolic blood pressure, *DBP* diastolic blood pressure

### Serum adiponectin

The percent change of the serum adiponectin level from the baseline to the final visit tended to be higher in the topiroxostat group, although the difference was not statistically significant (Topiroxostat: 3.9 %; 95 % CI −1.2 to 9.2 %, Placebo: −0.1 %, 95 % CI, −4.5 to 4.5 %; *P* = 0.2454).

### Safety

All AEs were designated and classified as mild to severe in terms of the severity by individual investigators, and their causal relationships with the study drug were evaluated. There were no deaths reported during the study. Serious AEs were reported in 2 patients (4 cases) from the topiroxostat group and 2 patients (2 cases) from the placebo group. In detail, “Polyarthritis (*n* = 1)” in the topiroxostat group, and “Acute hepatitis (*n* = 1)” in the placebo group were considered by the investigator to be related to the study drug, and patients with these AEs were withdrawn from the study.

The AEs that led to treatment withdrawal were “ALT, AST increased (*n* = 1)”, “Eczema (*n* = 1)”, and “Polyarthritis (*n* = 1)” in the topiroxostat group, and “Acute hepatitis (*n* = 1)” in the placebo group. Overall, the rate of AEs was similar in the two groups and the frequently reported AEs (≥5 %) are listed in Table 3. All of the AEs were mild to moderate in severity. The incidence of ‘alanine aminotransferase (ALT) increased’ was higher in the topiroxostat group than that in the placebo group. In detail, the incidence of concurrent increase of the total bilirubin or alkaline phosphatase with the ALT was similar in both groups (Table 4). In addition, the ‘ALT increased’ and ‘AST increased’ in the topiroxostat group were mild in severity in all cases.

**Table 3:** Summary of adverse events occurring in ≥5 % of patients in either treatment group (safety population)

AE	Number (%) of patients	
	Topiroxostat (*n* = 62)	Placebo (*n* = 60)
Any AEs	42 (67.7)	41 (68.3)
Nasopharyngitis	13 (21.0)	13 (21.7)
Conjunctivitis allergic	1 (1.6)	4 (6.7)
Rhinitis allergic	1 (1.6)	4 (6.7)
Upper respiratory tract inflammation	0 (0.0)	4 (6.7)
Diarrhea	1 (1.6)	3 (5.0)
Arthralgia	6 (9.7)	1 (1.7)
Gouty arthritis	9 (14.5)	5 (8.3)
ALT increased	8 (12.9)	0 (0.0)
Urine albumin present	0 (0.0)	3 (5.0)
AST increased	6 (9.7)	2 (3.3)

*AE* adverse event, *ALT* alanine aminotransferase, *AST* aspartate aminotransferase

**Table 4:** Liver function test results

	Number (%) of patients	
	Topiroxostat (*n* = 62)	Placebo (*n* = 60)
ALT ≥1.5 × ULN	11 (17.7)	3 (5.0)
AST ≥1.5 × ULN	5 (8.1)	3 (5.0)
Concurrent results		
ALT ≥1.5 × ULN and AST ≥1.5 × ULN	4 (6.5)	2 (3.3)
ALT ≥1.5 × ULN and total bilirubin ≥34.2 μmol/L	0 (0.0)	1 (1.7)
AST ≥1.5 × ULN and total bilirubin ≥34.2 μmol/L	0 (0.0)	1 (1.7)
ALT ≥1.5 × ULN and ALP ≥2.0 × ULN	1 (1.6)	0 (0.0)
AST ≥1.5 × ULN and ALP ≥2.0 × ULN	0 (0.0)	0 (0.0)

*ALT* alanine aminotransferase, *AST* aspartate aminotransferase, *ALP* Alkaline phosphatase, *ULN* upper limit of normal

## Discussion

To the best of our knowledge, this is the first clinical study to evaluate the effect of a xanthine oxidase inhibitor on the renal function under a double-blind condition. In this 22-week study, we set two primary efficacy endpoints, namely, the percent change of the serum urate from the baseline to the final visit and the change in eGFR from the baseline to the final visit. We showed that 22 weeks of treatment with 160 mg topiroxostat daily effectively reduced the serum urate level in patients with hyperuricemic CKD stage 3 with or without gout. Based on the results of previous reports, the urate-lowering efficacy of topiroxostat has been assumed to be mediated by XO inhibition [22, 23]. The achievement rate of a serum urate level of ≤356.88 μmol/L was 90.0 % in the topiroxostat group, but 0.0 % in the placebo group. The result in the placebo group was similar to that reported from the clinical study on febuxostat [20].

On the other hand, no statistically significant difference in the change of the eGFR from the baseline to the final visit was observed between the topiroxostat group and the placebo group. At the time of designing of the protocol for this study, there were no data on the changes of the eGFR induced by topiroxostat in CKD stage 3 patients. Therefore, we calculated the sample size for this study from the results of changes in the serum creatinine concentration in the allopurinol group and the control group observed at 6 months from baseline [10]. There could be various reasons for these results such as treatment environment. In retrospect, the different trend of the serum creatinine level between the control group in the allopurinol study and the placebo group in this study was observed. A 2-year study of allopurinol showed the amelioration of eGFR [11]. Therefore, the treatment period of this study was not sufficient for the evaluation of the change of eGFR. More adequate clinical study period and adequate sample size based on the results of the current study are needed, to evaluate the effect of topiroxostat on eGFR.

We obtained various results from the secondary efficacy endpoints in this study. We showed that the percent change of ACR from baseline to the final visit was approximately 30 % with time-dependent manner in topiroxostat group compared to placebo group. In addition, topiroxostat did not show the clear effect on either the change of blood pressure or the change of eGFR. The reported correlation between allopurinol and reduction of albuminuria is controversial. While one clinical study of allopurinol in patients with CKD suggested that allopurinol could have a potency to decrease albuminuria, another study reported no effect on albuminuria [10, 11]. On the other side, the finding of ACR-lowering effect by topiroxostat in this study is consistent with the findings of experimental studies of other xanthine oxidase inhibitors [24, 25]. In this study, we did not prohibit concomitant use of blood-pressure-lowering agents, including ACE inhibitors, ARBs, aldosterone blockers or renin inhibitors (RAA blockers). Also, it was not necessary for the patients to take maximal doses of the RAA blockers. Therefore, these results might have been affected by the different classes or doses of these drugs used concomitantly. To verify the robustness of the ACR-lowering effect of topiroxostat, we confirmed similar ACR-lowering effect in the other data set (per protocol set) in which the data of ACR after the time point were excluded if patients changed the type or dose of their blood-pressure-lowering agent during the study. Also, we considered the possible dependence of the degree of ACR reduction on the initial value. However, no relationship could be demonstrated between the baseline ACR and the change in the ACR in either group. In addition, the serum albumin levels in both groups remained stable during the study (data not shown).

The incidence of total AE was similar in both groups. The incidence of ‘ALT increased’ was statistically significantly higher in the topiroxostat group as compared with that in the placebo group. However, the frequency of concurrent increase of the ALT with the total bilirubin or alkaline phosphatase was similar in both groups. In this study, we excluded patients with hepatic dysfunction in exclusion criteria. Therefore, it will be important for physicians to monitor the liver function in clinical practice. The incidences of gouty arthritis or arthralgia were not statistically significantly different between the two groups, but tended to be higher in the topiroxostat group. In this study, we did not permit colchicine prophylaxis because of assessment of the onset of gouty arthritis in the patients. Also, the doses of topiroxostat were not increased in parallel with the level of serum urate in each subject. To minimize the incidence of gouty arthritis, anti-inflammatory prophylaxis and stepwise dose titration in accordance with the level of serum urate in each subject need to be considered. In addition, it is important to educate the patients is necessary to make them aware of the possible onset of gouty arthritis during the initiation of urate-lowering therapy [5].

The limitations of this study are as follows, first, the study period was short to assess the change of eGFR from baseline to the final visit. Second, ACR was used instead of measuring the urinary albumin excretion, because 24 h urine collection would have been difficult in outpatients; this study was conducted as a multicenter study, and the ACR has been shown previously to be positively correlated with the urinary albumin excretion [26]. Also, the ACR was not calculated by the levels of albumin and creatinine in the first morning void urine. Third, we did not measure the true GFR and ambulatory blood pressure monitoring, because of the difficulty in performing these measurements in the outpatient setting.

In the next step, we consider that additional clinical studies are needed to validate the effect on albuminuria of topiroxostat in patients with high albuminuria levels, because the percent change of the ACR from the baseline to the final visit in this study was set as a secondary endpoint and the baseline level of ACR was not sufficiently higher in the both groups. Also, it is important to clarify the maximum effect on albuminuria in clinical practice, its time relationship, and dose-responsiveness.

In conclusion, the results of this study demonstrated that treatment with topiroxostat effectively reduced the serum urate levels in Japanese hyperuricemic patients with renal impairment, with or without gout. In addition, topiroxostat also exhibited the potential to decrease the albuminuria in these patients, although further clinical studies are needed to validate its efficacy.
